# Identification of a novel and potent small molecule inhibitor of SRPK1: mechanism of dual inhibition of SRPK1 for the inhibition of cancer progression

**DOI:** 10.18632/aging.202301

**Published:** 2020-12-03

**Authors:** Anshuman Chandra, Hanumappa Ananda, Nagendra Singh, Imteyaz Qamar

**Affiliations:** 1 School of Biotechnology, Gautam Buddha University, Greater Noida, U.P. 201312, India; 2 Department of Clinical Embryology, Kasturba Medical College, Manipal Academy of Higher Education, Manipal, Karnataka 576104, India

**Keywords:** splicing inhibitor, structure based drug design, flow cytometery, MTT assay, virtual screening

## Abstract

Protein kinases are the family of attractive enzyme targets for drug design with relevance to cancer biology. Serine arginine protein kinase 1 (SRPK1) is responsible for the phosphorylation of serine/arginine (SR)-rich proteins. Alternative Splicing Factor/Splicing Factor 2 (ASF/SF2) involved in mRNA editing. ASF/SF2 is over expressed in many cancers and plays crucial roles in the cell survival. Phosphorylation of ASF/SF2 is decisive for its functions in cancer. In search of potential anticancer therapeutic agents for attenuating phosphorylation of ASF/SF2, we have explored specific and potential inhibitors of SRPK1 from natural and drug like compounds databases using *in-silico* methods. Compound ZINC02154892 (C02) was found to be the most potent inhibitor for SRPK1. *In-vitro* molecular and cell biology studies have shown C02 as a potent and specific inhibitor of phosphorylation of ASF/SF2 and cell survival in leukemic cell line. Structural analysis of SRPK1 with compound C02 revealed a unique pattern of binding targeting ATP binding site along with inhibiting recruitment of ASF/SF2 by SRPK1. The possibilities of compound C02 to be used as a lead compound paving way for the development of potent and specific inhibitors of SRPK1 for designing of novel potential anticancer inhibitor is inferred from the current studies.

## INTRODUCTION

Serine/arginine (SR)-rich proteins are a well known family of splicing factors with the function of removal of intervening sequences from primary transcript and production of a large number of mRNA isoforms by processes known as constitutive and alternative splicing [1, 2]. In eukaryotes, alternative splicing contributes to the complexity of proteome and regulates protein functions. Besides splicing, SR proteins also participate in other cellular process such as transcription, mRNA transport, stability and protein translation [3]. Differentially expressed gene levels of splicing regulatory factor have been observed in the progression of various cancers, affecting the splicing patterns of many genes that function in certain cancer-specific biological pathways, including cell cycle progression, cellular proliferation, migration and processing [4].

ASF/SF2, encoded by *Serine / arginine-rich splicing factor 1* (*SRSF1*) is a member of the highly conserved SR protein family and plays a vital role in cell viability and cell cycle progression [5]. ASF/SF2 has a modular structure with the presence of two RNA recognition motifs (RRMs) at their N terminal and a RS domain rich in serine and arginine (RS) residues at their C terminal [6]. The RRM and the RS domain of ASF/SF2 protein are functionally independent of each other. Unlike other SR proteins, the RS domain of ASF/SF2 that is shorter and bifurcated contains a repeat of eight consecutive RS dipeptides (RS 1 motif) followed by short stretch of RS dipeptides (RS 2 motif). This RS domain of ASF/SF2 is extensively phosphorylated in the cytoplasm and exists in differentially phosphorylated (hypo- and hyperphosphorylated) states for its sub-cellular localization and functions [7].

The Serine/ arginine-rich protein kinase-1 (SRPK1) a member of SRPK family and Cdc-2 like (Clk/Sty) kinases predominantly phosphorylate ASF/SF2 in cytosol and nucleus respectively. SRPK1 was identified in 1994 as a highly RS-specific protein kinase which phosphorylate all the serine residues present within the RS domain of ASF/SF2 with very high specificity and efficiency [8]. Phosphorylation of RS domain of ASF/SF2 by SRPKI enables its localization from cytoplasm to the nucleus and mediates its nuclear import and recruitment to nuclear speckles, where it is further phosphorylated by Clk/Sty [9].

SRPK1 also regulates splicing of pro-angiogenic VEGF-A1, a crucial factor of neovascular eye diseases, through phosphorylation of SRSF1, enabling SRSF1 nuclear translocation and binding to the proximal splice site in VEGF-A pre-mRNA [10].

Recent studies have proposed that ASF/SF2 protein levels vary widely among different cell types [5]. Tight controlled expression of ASF/SF2 appears significant in normal cellular physiology [11–13]. It has been identified as an oncoprotein involved in many types of human cancers, including those of the lung, colon, breast, hepatocellular and pancreatic carcinoma [12–14]. Over expression of ASF/SF2 is sufficient to cause transformation of fibroblast by controlling alternative splicing of tumor suppressors and oncogenes [15]. The accumulated knowledge about ASF/SF2 provides critical insight into the integral roles it plays in maintaining cellular functioning and as new targets for anti-cancer treatment.

Several reports on designing of synthetic antagonists against SRPK1 are available [16–21]. Most of these compounds recognize ATP binding site in SRPK1. Owning to the fact that most inhibitors interact with the highly conserved ATP binding clefts of kinases and other cellular enzymes, it is a daunting challenge to develop therapeutic drug that targets only SRPK1. Therefore, we explored potential compounds from natural and drug like database, which can bind to overlapping sites beyond ATP binding on SRPK1 to specifically interrupt its interactions with its substrate, ASF/SF2 and also block ATP hydrolysis required for activation. A high-throughput structure-based virtual screening (SBVS) was carried out in which docking scores were used to characterize the novel lead compounds. Chemical stability of top compounds were screened and the most stable and potent compounds were used for further physiochemical and pharmacokinetics analysis. The best compounds were tested *in vitro* using leukemic cell lines by performing cytotoxicity assay and western blotting using anti SR proteins antibodies. Moreover the mechanism of apoptosis was also determined by qRTPCR. This study provided novel specific inhibitors of SRPK1 from library of natural compounds database, which may be useful in the development of safe and effective anti-cancer agents.

## RESULTS

### Structure based virtual screening

More than a million compound structures were used in Virtual screening procedure. The prepared 3D structures of compounds were docked into the generated grid containing active site of SRPK1 and its residues interacting with ASF/SF2. The step filtering process present in virtual screening workflow resulted in 11912 compounds for critical docking (XP). Top 500 compounds were chosen for further analysis on the basis of their scores.

Prediction of factors like absorption, distribution, metabolism and excretion of the lead compounds was done to improve the success rate of lead optimization and testing. The top compounds have donor Hb ranging from 2 to 5 and Hb acceptor from 4.25 to 10, Mol wt was reported to be less than <550, QPlogPo/w is < 5 to ensure compliance with the Lipinskis rule of five. The compounds which were in permissible range of Lipinski rule were considered for drug likeliness properties (Supplementary Table 1).

### Docking studies

**Figure 1:**
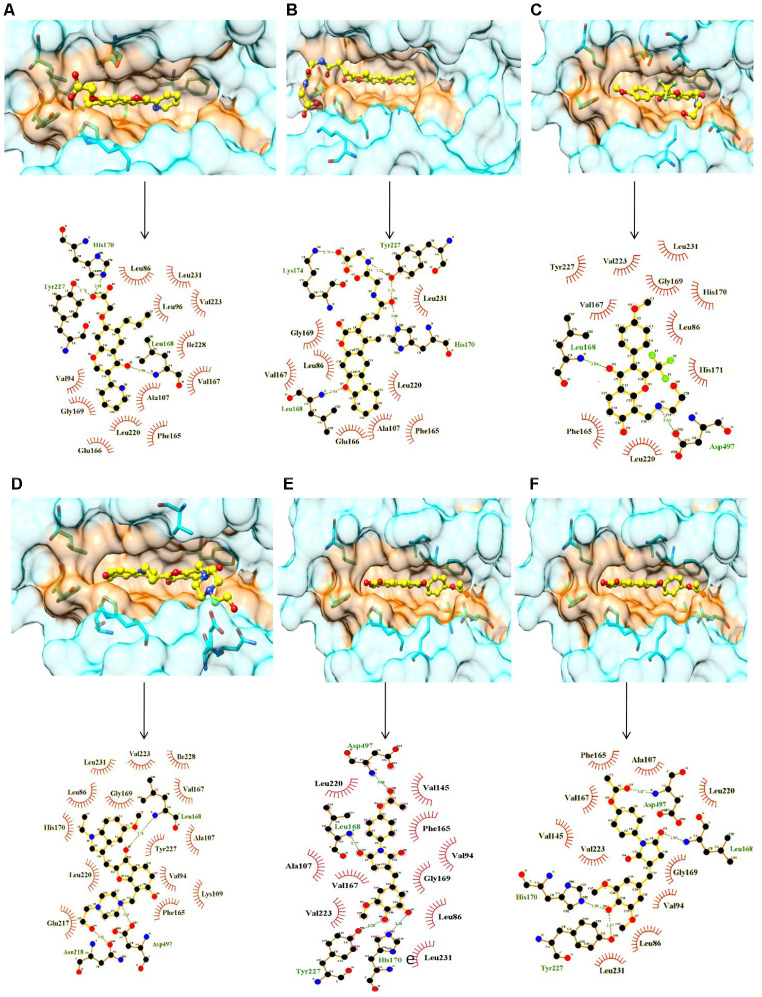
**Surface and ligplot diagrams showing docking and binding interactions of selected compounds at the ATP site of SRPK1.** (**A**) Compound C01, (**B**) Compound C02, (**C**) Compound C03, (**D**) Compound C04, (**E**) Compound C05, (**F**) Compound C06. All compounds occupied the similar space in the binding site. It is noteworthy to observe the additional unique Interactions of compound C02 with His170, Tyr227 and Lys174 amino acid residues of SRPK1.

The manual analysis was used for filtration of best pose of each compound and the top six compounds which were chemically stable were considered. The compounds which were acid/base labile or undergo hydrolysis at high or low pH were also filtered out. The compounds having significant difference in Glide energy, E-model and XP GScore of reference compound SRPIN340 were selected. Finally six compounds were chosen for analysis. All these six best compounds belong to natural ZINC database (Table 1). For the ease of reporting these compounds are given numbers from C01 to C06 as ZINC00518605 (C01), ZINC02154892 (C02), ZINC23127139 (C03), ZINC28182826 (C04), ZINC62001834 (C05), ZINC70666371 (C06). All six compounds were docked considering ATP binding groove along with ASF/SF2 interaction sites on SRPK1 (Figure 1). *In silico* results indicated that all six compounds have good affinities with SRPK1. Compounds C01, C03, C04, C05 and C06 bind at the ATP binding site whereas compound C02 extended its binding to additional regions and expected to have higher selectivity towards interrupting the SRPK1-SF2/ASF complex formation.

**Table 1:** SRPK1 binding properties of the screened natural compounds and known inhibitors.

**S.No.**	**Compounds**	**Residues involved in polar interactions**	**Residues involved in non-polar interactions**	**Glide e-model**	**Glide energy**	**XP GScore**
1.	**Compound C01 (ZINC00518605)**	Leu168, His170, Tyr227	Leu86, Val94, Leu96, Val145, Phe165, Val167, Leu168, Leu220, Tyr227, Ile228 and Leu231	-72.51	-54.61	-11.31
2.	**Compound C02 (ZINC02154892)**	Leu168, His170, Lys174, Tyr227	Leu86, Val94, Ala107, Val145, Phe165, Val167, Leu220, Leu231, Ala496	-92.63	-60.29	-12.40
3.	**Compound C03 ZINC23127139**	Leu168, Asp497, His170	Val94, Lue96, Ala107, Lys109, Val145, Phe165, Val167, Leu220 Leu231, Val223, Tyr227, Ile228, Ala496	-80.70	-47.34	-12.01
4.	**Compound C04 ZINC28182826**	Leu168, Asn218, Asp497	Leu86, Val94, Leu96, Ala107, Lys109, Val145, Val167, Phe165, Lys215, Leu220 Val223, Ile228, Leu231, Tyr227, Ala496	-75.41	-46.37	-11.34
5.	**Compound C05 ZINC62001834**	Leu168, Asp497, Tyr227	Leu86, Val94, Leu96, Ala107, Leu128, Val145, Phe165, Val167, Leu220, Val223, Ile228, Leu231, Ala496, Leu498	-74.92	-47.52	-12.46
6.	**Compound C06 (ZINC70666371)**	Leu168, Tyr227, Asp497	Leu86, Val94, Leu96, Leu128, Val145, Phe165, Val167, Leu220, Val223, Leu231, Ala496, Leu498	-72.51	-45.24	-11.13
7.	**SRPIN340 [17]**	Leu168	Leu86, Ala107, Val145, Leu168, Phe165, Val167, Val223, Tyr227, Leu231	- 75.89	- 47.86	- 9.61
8.	**SPHINX31 [14]**	Leu168, Lys109	Leu86, Trp88, Gly89, Ser92, Val94, Ala107, Phe165, Glu166, Val167, Gly169, His170, Leu220, Ala496 and Asp497	-	-	-
9.	**Compound I [14]**	Leu168	Leu86, Tyr88, Gly89, Ser92, Val94, Ala107, Phe165, Glu166, Val167, Gly169, His170, Leu220, Ala496 and Asp497	-	-	-
10.	**Alectinib [24]**	Leu168	Arg84, Leu86, Val94, Ala107, Phe165, Val167, His170, Leu220, Val223, Tyr227, Leu231	-	-	-

To ensure the effectiveness of the present study, SRPIN340 [18], a known SRPK1 inhibitor was taken as control. Docking results of the control protein-ligand complex showed a stable interactions with Glide energy, E-model and XP GScore of 47.86, - 75.89 and - 9.61 respectively. These scores were taken as minimum cutoff for selection of virtual hits from natural and drug like compound database of ZINC12 [22].

### Molecular dynamics studies

### Root mean square deviation (RMSD)

In order to evaluate the stability of SRPK1 and its complexes, the RMSD value for the C [α] backbone was calculated for 100 ns simulations from the equilibrated structure. The stability of the protein relative to its conformation can be driven by the deviations generated during the course of its simulation. More stable protein structures are indicated by smaller deviations. Supplementary Figure 1A gives a clear idea that free SRPK1 and its complexes demonstrated different patterns of RMSD, but all the systems got the equilibration state in last 40 ns. Apo-SRPK1 showed an average RMSD value of 0.313 nm while reference and other compounds SRPIN340, C01, C02, C03, C04, C05, and C06 showed RMSD values of 0.283, 0.279, 0.35, 0.285, 0.232, 0.322 and 0.317 nm respectively. The SRPK1-C02, SRPK1-C05, and SRPK1-C06 demonstrated slightly higher deviation than the Apo-SRPK1. This magnitude of fluctuation, together with a minimal difference in the average RMSD value, led to the inference that the simulation produced stable trajectories. In terms of RMSD, the SRPK1-C03, SRPK1-C04, SRPK1-SRPIN340, SRPK1-C01 showed similar values while SRPK1-C02, SRPK1-C05 and SRPK1-C06 showed a slightly higher value. All the calculations were done for the last 40 ns of stable trajectory.

### Root mean square fluctuations (RSMF)

To examine the effect of the fluctuation on whole protein and also on individual residues, the RMSF of C [α] atoms were examined for all the systems. RMSF imparts data on the consequence of ligand binding on the residue-wise mobility of all the Apo-SRPK1 and SRPK1-complexes. The RMSF determined that the SRPK1-complexes indicated undoubtedly lower fluctuations than the free SRPK1 throughout the structure (Supplementary Figure 1B). Complexes of SRPK1 with SRPIN340, C01, C02, C03, C04, C05, and C06 showed RMSF values 0.12, 0.12, 0.116, 0.131, 0.108, 0.113 and 0.113 nm respectively. During binding, all the ligands showed less fluctuation than Apo-SRPK1 while, C03 caused more fluctuation as compared to all other ligands. The results suggested that the ligand binding led to reduced fluctuation in the protein active site to stabilize the complex.

### Radius of gyration (Rg)

The conformational geometries of the protein-compounds complexes were recorded by examining the Rg values. The Rg value was calculated for the last 40 ns simulation trajectories. The average Rg value for free SRPK1 and its complexes with SRPIN340, C01, C02, C03, C04, C05, and C06 was found to be 2.12, 2.19, 2.14, 2.11, 2.15, 2.13, 2.15 and 2.19 respectively. The Rg displayed comparable pattern till the end of simulation, for all the complexes. Since Rg describes the solidity of the structure, results explains that there was no significant alteration in the hydrodynamic gyration of the protein structure upon ligand binding. SRPK1-C01, SRPK1-C02, SRPK1-C03, and SRPK1-C04 showed lower Rg value as compared to the known inhibitor SRPIN340. It is evident that SRPK1-C02 complex is slightly comparatively more stable than the other complexes (Supplementary Figure 1C).

### Hydrogen bonds

Hydrogen bonding is one of the most important interaction for stabilizing the protein-ligand complexes. The hydrogen bonds were analyzed for all the complexes using last 40 ns trajectories. SRPK1 complex with C02, C03 and C06 showed more number of hydrogen bonds as compared to the other complexes. The average number of hydrogen bonds in SRPK1-SRPIN340, SRPK1-C01, SRPK1-C02, SRPK1-C03, SRPK1-C04, SRPK1-C05, and SRPK1-C06 were 0-1, 0-1, 0-4, 0-4, 0-3, 0-3 and 0-4, respectively during simulations. The results suggested that the compounds C02, C03, C04, C05, and C06 had stronger binding with SRPK1 during the course of simulation leading to stabilization of the complex (Supplementary Figure 1D).

### Principal component analysis (PCA)

The PCA reflects the overall expansion of a protein during different simulations. The dynamics of SRPK1 was calculated using gmx covar module with respect to the backbone. The sum of the eigen values is a measure of the total motility in system. It can be used to compare the flexibility of a protein under different conditions. The Supplementary Figure 1E represents the eigenvalue in decreasing order versus corresponding eigenvector for free SRPK1, SRPK1-SRPIN340, SRPK1-C01, SRPK1-C02, SRPK1-C03, SRPK1-C04, SRPK1-C05, and SRPK1-C06 complexes. From the 50 eigenvectors, it was observed that the first ten eigenvectors accounted for 80.13, 74.27, 80.24, 70.86, 77.35, 75.52, 73.73, 71.09% for Apo-SRPK1, SRPK1-SRPIN340, SRPK1-C01, SRPK1-C02, SRPK1-C03, SRPK1-C04, SRPK1-C05, and SRPK1-C06 respectively. The reference compound showed less motion as compared to other complexes. It was found that SRPK1-C01, SRPK1-C03 and SRPK1-C04 showed higher motions than the control SRPIN340. From the PCA, we can conclude that SRPK1-C02, SRPK1-C05, and SRPK1-C06 were so far the best-studied complexes (Supplementary Figure 1F).

### Gibbs free energy

The eigenvectors PC1 and PC2 were used to plot gibbs free energy landscape using g_covar, g_anaeig, and g_sham tools in gromacs. The landscape depicts the local minima during the simulation trajectory. The Apo-SRPK1, SRPK1-SRPIN340, SRPK1-C01, SRPK1-C02, SRPK1-C03, SRPK1-C04, SRPK1-C05, and SRPK1-C06 complex 2D plot is represented in Supplementary Figure 2. Lower energy is indicated by deep blue color. Each plot indicates different patterns of global free energy minima. Hence, it is clear that after ligand binding SRPK1 gains a different yet stable conformation. The most metastable conformational states were seen in the binding of SRPIN340, C02, C05, and C06 to SRPK1, in which local minima distributed to about two to three regions within the energy landscape. The Free SRPK1 formed one metastable conformation during the whole population of trajectories.

### Binding free energy

The binding free energy calculations were Carried out for each SRPK1-ligand complex considering only the last 20 ns of simulation [23]. MM-PBSA [24, 25] method was used for calculation. The total binding free energy was -152, -110, -172, -183, -150 and -182 kJ/mol for SRPK1-C01, SRPK1-C02, SRPK1-C03, SRPK1-C04, SRPK1-C05, SRPK1-C06 and SRPK1-SRPIN340 Complexes respectively (Supplementary Table 2).

### Cytotoxicity assay

All six compounds were screened for their ability to induce cytotoxicity in cancer cell lines Jurkat, A549, K562, and HeLa. MTT assay showed that treatment with different concentrations of the compounds (48 h) resulted in cell death at varying levels. compound C02 showed maximum inhibition on cell proliferation, followed by compound C03 (Table 2), The IC50 of compound C02 was determined to be 9.51, 29.76, 25.81 and 34.53 μM in Jurkat, A549, K562 and HeLa cells respectively. Trypan blue assay showed reduced number of live cells following treatment with compound C02 and MTT assay confirmed the reduction in cell proliferation. In order to check the mechanism of action of compound C02, Jurkat cells were selected for further assays.

**Table 2:** Inhibition studies of the selected compounds: Anti-proliferative properties of potential lead compounds (C01-C06) on Jurkat, A549, K562 and HeLa cells.

**Name of the compound**	**Cell lines (IC_50_ value in μM)**			
	**Jurkat (T cell leukemia)**	**A549 (Adenocarcinoma)**	**K562 (Myeloid leukemia)**	**HeLa (Cervical cancer)**
C01	> 50	> 50	> 50	> 50
C02	9.51	29.76	25.81	34.53
C03	> 29.38	44.16	40.61	> 50
C04	> 50	> 50	> 50	> 50
C05	> 50	> 50	> 50	> 50
C06	> 50	> 50	> 50	> 50

IC50 values were based on MTT assay results at 48 h time point.

### Compound C02 causes the accumulation of the cells in the SubG1 phase of the cell cycle

**Figure 2:**
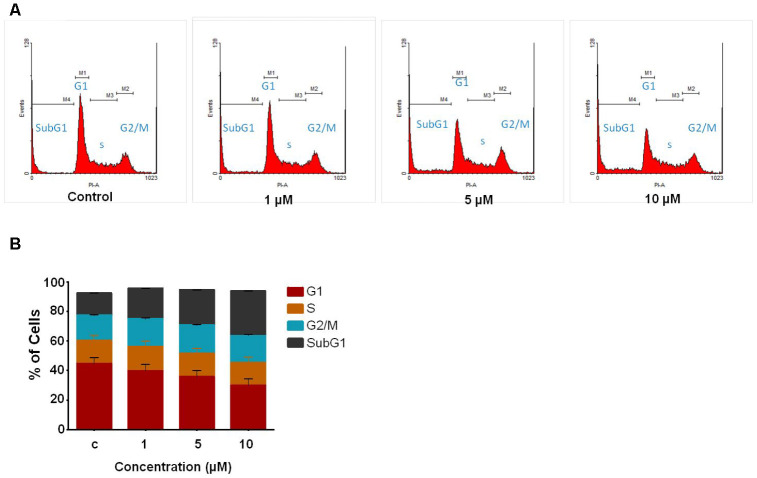
**Study of cell cycle analysis of Jurkat cells following C02 compound treatment.** (**A**) Jurkat cells treated with C02 compound (1, 5 and 10 μM) for 48 h, harvested and stained with propidium iodide and subjected to flow cytometry. (**B**) Histograms obtained after FACS analysis of C02 treated Jurkat cells.

Following preliminary examinations of cytotoxicity assays, we further tested the effect of compound C02 cell cycle progression in Jurkat cells by flow cytometric analysis at 48 hr. Upon addition of C02 (1, 5 and 10 μM) a remarkable change was observed in the cell cycle distribution particularly in SubG1 population at 10 μM. The results showed notable accumulation of SubG1 population (Figure 2), suggesting that C02 promotes cell death through apoptosis. The percentage of cell populations in each phase of cell cycle is represented in bar diagram (Figure 2B).

### Effect of compound C02 on SR protein phosphorylation

**Figure 3:**
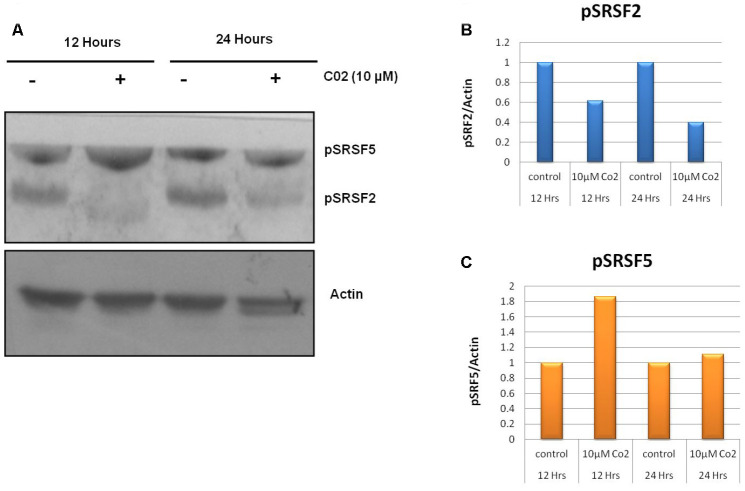
**Effects of compound C02 on the SRPK1 mediated phosphorylation of SR proteins.** (**A**) Western blotting analysis after treatment with compound C02 (10 μM) and negative control (DMSO) for 12 and 24 h on Jurkat cells is shown. SR protein phosphorylation was detected using mAb1H4, which recognizes phosphorylated serine arginine epitopes present on SR factors. The blot was re-probed with actin to be used as endogenous control. (**B**, **C**) The protein bands were also determined relative to the endogenous control Actin by using densitometry software.

Post translational modifications of SR proteins are an important event for aberrant splicing in cancerous cells. To evaluate the effect of lead compound C02 on phosphorylation of SR proteins SRSF2 and SRSF5, western blot analysis were performed after the treatment of C02 on Jurkat cells. Upon addition of C02 (10 μM), a significant decrease in the phosphorylation of level of SRSF2 was observed at 12 h and also 24 h (Figure 3). However inhibition of SRSF5 phosphorylation was not altered significantly. This shows that compound C02 is associated with specific inhibition of SRPK1 mediated phosphorylation of ASF/SF2 (SRSF1) in Jurkat cells.

### Mechanism of apoptosis in Jurkat cells by compound C02

**Figure 4:**
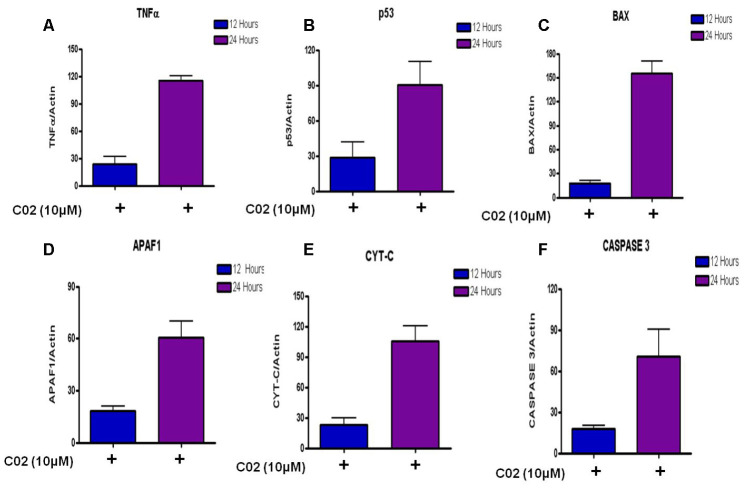
Relative expression of apoptotic factors determined by quantitative real time PCR (qRT-PCR) analysis of (**A**) Tumour necrosis factor-α (TNF-α), (**B**) Tumour suppressor (P53), (**C**) Apoptosis regulator (BAX), (**D**) Apoptotic protease activating factor 1 (APAF 1), (**E**) Cytochrome-c and (**F**) Caspase 3 genes in Jurkat cells treated with 10μm Compound C02 for 24 h. mRNA levels of apoptotic factors were determined relative to the endogenous control Actin, according to the formula 2 to the power of delta cycle threshold (2DCt), where DCt¼Ct, reference gene – Ct, test gene. Differences between experimental groups were tested for significance using nonparametric Mann–Whitney test (GraphPad Prism version 5, San Diego, CA), for both mRNA, protein expressions and other analysis. Levels of significance are indicated by p< 0.05.

To determine the apoptotic mechanisms under the effect of Compound C02 on Jurkat cells, selected genes belonging to apoptosis activator (Supplementary Table 3) were assessed by qRT-PCR analysis. The jurkat cells were treated with 10 μM of Compound 02 at two different time points 12 h and 24 h. The apoptotic effect of C02 was particularly strong on 24 hours of treatment with 10μM concentration in Jurkat cells. Apoptosis regulator genes of the intrinsic apoptosis pathway BAX were observed to be overexpressed (10 fold at 24 hours) and triggered the release of Cyt-C (5 fold at 24 h) from mitochondrial membranes. An increase in the expression of APAF 1 (3 fold) and activated caspase 3 (3 fold) was also detected at 24 hr of 10 μM C02 treatment. Additionally, the TNF-α, tumour suppressor P53 (3 fold) and cytochrome-c (5 fold) were also found overexpressed (Figure 4). Thus it can be concluded that the Jurkat cells treated 10 μM of Compound 02 activates the intrinsic pathway of apoptosis.

### Analysis of binding of compounds to SRPK1

SRPK1 binds ATP at the typical nucleotide binding site located as a surface cavity in order to perform kinase functions. The ATP binding site is mainly formed by residues Val167- His171, Ser92, Val94, His90, Phe91, Leu220 as can be inferred from the reported structure of complex with SRPK1 [9]. Reported synthetic compound I [16], SRPIN340 [19] and Sphinx31 [16] binds at the same site making attractive interactions with SRPK1. Whereas, a tyrosine kinase inhibitor alectinib recognizes slightly different but overlapping space of the ATP binding site of SRPK1 [26].

All selected six compounds along with the reference inhibitor SRPIN340 were observed to be located in the ATP binding site of SRPK1 with variable attractive interactions (Table 1). The carboxylic group of C01 formed H-bond with the side chain of Tyr227 along with a salt bridge with His170. One additional H-bond with the main chain NH of Leu168 with C01 was also observed. C01 is also surrounded in by hydrophobic cavity made by side groups of Leu86, Val94, Leu96, Ala107, Val145, Phe165, Val167, Leu220, Val223, Tyr227, Ile228 and Leu231 amino acids of SRPK1 (Figure 1A). C02 has gained one extra salt bridge with the side chain of Lys174 and an H-bond with Tyr227 whereas hydrophobic interactions with Val223, Leu231 and Ile238 are lost in comparison to C01 (Figure 1B). The orientation of C03 is stabilized by presence of two H-bonds each with the main chain NH of Leu168 and side group of Asp497 along with van der Waals interactions in the cavity of the enzyme (Figure 1C). C04 has occupied more space and interacted with 2 extra H-bonds with Asn218 and Asp497 residues in comparison to the binding of compound C01 (Figure 1D). C05 makes H-bonds with the Leu168, His170, Tyr227 and Asp497 residues. Both C01 and C05 shared common van der Waals interactions with SRPK1 (Figure 1E). As per structural analysis, C01 and C02 are the only who make additional salt bridges attractive interactions with SRPK1 and may have highest chemical affinities with the enzyme. It is also clear that polar/charged interactions with the side chains His170 and Tyr227 plays central roles in maximizing binding affinities of ligands to SRPK1. These interactions are absent in case of earlier reported inhibitors.

**Figure 5:**
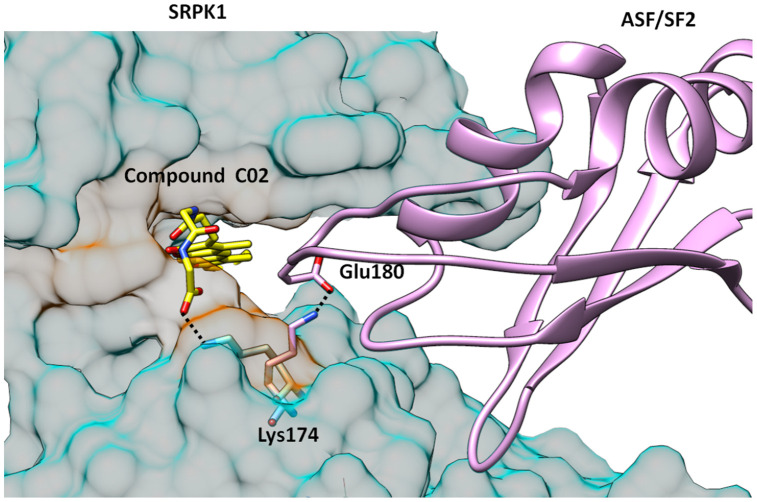
**Structure of SRPK1-compund C02 complex is superimposed with SRPK1-ASF/SF2 complex. **It is clearly seen that C02 has interrupted the complex formation by engaging Lys174 which forms critical attractive interaction with Glu170 of ASF/SF2. Lys174 is rotated almost 2-folds towards ATP binding site and is unavailable to form salt bridge interactions with Glu170 of ASF/SF2.

Compound C02 has also gained one extra salt bridge interaction with the side chain of Lys174. It is noteworthy that Lys174 is involved formation of ASF/SF2-SRPK1 complex by making ionic interaction with the side group of Glu184 of ASF/SF2, which is one of the key interactions for stability of the complex and to bring substrate ASF/SF2 close to the ATP binding site for phosphorylation. In order to interact with carboxylate group of compound C02, Lys174 acquired almost two fold rotation and over 6Å shift of side chain amino group from the orientation it binds to Glu184 of ASF/SF2 in the complex formation (Figure 5), Which is an additional feature of compound C02 in order to interrupt productive binding of ASF/SF2 to SRPK1. This additional ionic interaction is ascribed to the presence of Gly-Gly dipeptide in the structure of compound C02. Therefore, compound C02 may have possible roles in inhibiting binding of both ATP as well as recruitment of ASF/SF2 by SRPK1.

### Comparison of binding with other SRPK1 inhibitors

Reported synthetic inhibitors SRPIN340, compound I, sphinx31 and alectinib share same space in the ATP binding site of SRPK1. All the six compounds in present study also recognize same site with variable positioning in SRPK1. These compounds were selected on the basis of docking scores higher than SRPIN31, which was used as reference compound during *In silico* analysis. It is evident from the structural analysis of SRPK1 complexes that all the six compounds make more number of attractive interactions than SRPIN340 with SRPK1. In fact SRPIN340, sphinx31, compound I and alectinib make only one H-bond with the main chain NH atoms of Leu168, which is also common with other ligands binding (Table 1). Whereas, presently reported compounds make many more polar interactions in form of H-bonds and salt bridges than all other existed inhibitors of SRPK1. Only hydrophobic interactions with ATP binding cavity are common in all the compounds. The binding of compound C02 is exclusive in all known inhibitors as it recognizes additional residues along with binding to the common ATP site on SRPK1, that is also responsible for higher specificity of compound C02.

## DISCUSSION

SRPK1 is involved in tumor growth in case of several cancers and considered to be among attractive targets for the development of anti-cancer therapeutic compounds. The available designed inhibitors of SRPK1 recognize and fit into the ATP binding site. As ATP binding site is common in all kinases and other enzymes, the approach of targeting ATP binding site alone may not yield specific inhibitors of SRPK1, which may lead to binding to several other physiological targets resulting in non-desirous adverse physiological effects. Therefore, it is of great importance to find more selective inhibitors of SRPK1. Reported *In silico* studies have inferred that that all the selected six compounds under study have good affinity for SRPK1. Whereas, only compound C02 has shown considerable effects on cancer cell viability in Jurkat cells (T-cell leukemia). Additionally, compound C02 was also found to be inhibitor of cell growth with a dual mechanism, as it inhibits ASF/SF2-SRPK1 complex formation along with ATP binding to SRPK1, which provide more selectivity to compound C02. Similarly, the phosphorylated form of pSRSF2 was also significantly decreased on treatment with compound C02. This suggest that the compound C02 is an effective inhibitor of phosphorylated form of pSRSF2. In addition, compound C02 triggers intrinsic pathway for apoptosis in Jurkat cell lines via Activation of activation of apoptotic factors. Therefore, it also may be concluded from the structure analysis that specifically targeting His170, Tyr227 and Lys174 in addition to common interactions with ATP binding site are desired for the designing of potent and selective inhibitors of SRPK1 to specifically interrupt SRPK1-ASF/SF2 complex formation along with ATP binding. Furthermore, ADME evaluation of the compound C02 showed its chemical behavior among a series of parameters that may enhance its drug likeness properties. *In-silico* binding affinities of all the six compounds were higher than the reference compound SRPIN340, which is also attributed to the more number of attractive polar interactions than existed compounds with SRPK1 (Table 1). It is also evident that compound C02 inhibit SRPK1 activity *In vivo* leading to disconcerting the sub cellular localization of ASF/SF2 and adversely affecting the cellular viability. It may also be inferred that compound C02 is more likely to provide new leads for the development of the novel and specific drugs against cancer by specifically inhibiting SRPK1 and its association with substrate ASF/SF2.

## MATERIALS AND METHODS

### Accession of target protein and ligands

The crystal structure coordinates of target protein (SRPK1), protein-inhibitor complexes and protein-substrate complex (PDB Id: 1WAK, 4WUA, 3BEG) were downloaded from the PDB database [27]. Structures of small organic compounds were obtained from ZINC12 Database [22]. The Natural compound library and Drug-like compound library were selected for the study. 728,747 compounds were downloaded in 2-dimensional SDF format from the Zinc database.

### Control validation

The ability of the docking to identify and screen novel SRPK1 inhibitors from selected library was tested by applying the mentioned docking procedure for SRPIN340 [18] that obtained from co-crystal structure of SRPK1 complex with its known inhibitor (PDB: 4WUA).

### Structure based virtual screening

SBVS is an important computational tool used to screen large number of compound in chemical library for identifying specific and potential virtual hits. SBVS eventually reduce the compounds to a manageable number for their biological evaluation against the target protein and now has fundamental part of contemporary drug research. Virtual screening workflow present in Schrodinger suit was deployed. A multi-step filtering method was used in which top 10% of resulted compounds are subjected to subsequent screening step. A total of 12,40,389 conformers were screened sequentially in High throughput Virtual Screening (HTVS), Standard Precision (Glide SP) and Glide XP (Extra Precision). (Supplementary Data).

### ADME prediction

Prior ADME prediction Prevents the failure of lead drug candidate in later trial phases of drug development. Hence, the ADME properties are considered critical in the process. Prediction of ADME properties of the compounds was carried out using Qikprop [28]. Further physicochemical properties like cell and skin permeability, absorption etc. and pharmacokinetic relevant properties like hydrogen bond donor and acceptor, partition coefficient were also predicted. The compounds filtered based on the Lipinski’s rule for further analysis.

### Molecular dynamics simulations

Molecular dynamics (MD) simulations were performed in order to study the conformational dynamics of protein and its binding to top six compounds. Gromacs 4.5.6 package [29] was used for MD simulations of apo, inhibitor control and the best pose of ZINC00518605 (Compound C01), ZINC02154892 (Compound C02), ZINC23127139 (Compound C03), ZINC28182826 (Compound C04), ZINC62001834 (Compound C05), and ZINC70666371 (Compound C06). All system were subjected to 100 ns time period MDS studies, for predicting stability of apo-SRPK1 and other SRPK1-ligand bound systems. (Supplementary Data).

### MTT assay

Cytotoxic effect of the identified compounds against the human T cell lymphoblast-like cell line (Jurkat), lung carcinoma (A549), myelogenous leukemia (K562) and cervical cancer (HeLa) cells (5x10^4^ cells/well) was assessed using 3-(4, 5- dimethyl-2-yl02, 5-diphenyl tetrazolium bromide (MTT) assay. The test compounds (C01, C02, C03, C04, C05 and C06) were dissolved separately in DMSO and the cells were treated with different concentrations of compounds (1, 10, 25 and 50 μM). Cells in the control wells received the same volume of medium containing DMSO. After 48 h treatment, cells were harvested and incubated with MTT (0.5 μg/mL) for 3 h at 37° C in 96 well plate. The blue MTT formazan precipitate formed by the viable cells was solubilized by addition of 100 μl DMSO. The suspension was placed in microvibrator for 5 minute and absorbance was measured at 540 nm using multimode reader (Varioskan Flash Multimode, Thermo scientific, USA). The experiment was performed in triplicate and repeated at least three times [19].

### Trypan blue dye exclusion assay

To determine the growth inhibitory activity of the compounds, 0.5 × 10^5^ cell/ml were plated in 24 well plate (Corning, USA.) in 1ml of complete medium and treated with various concentration of the compounds (1, 10, 25 and 50μM) of individual compounds. The cells were stained with 0.4% trypan blue and counted on a hemocytometer after treatment with compounds at 48 hours time point as described.

### Cell cycle analysis

As C02 was observed to be the best compound to inhibit cancer cell growth therefore it was used for further investigations. Jurkat cells were seeded in 24 well plate at 0.5 x 10^5^ cells/ml of complete growth culture media, incubated for 24 h. After incubation, the cells were treated with different concentrations of compound C02 (1, 5 and 10 μM). After 48 hours, the cells were harvested, washed with 1 X PBS and then fixed in 70% ethanol at 4° C overnight. Before acquiring Flow cytometry data, cells were washed with 1 X PBS, and resuspended in 300 μl of 1 X PBS. RNAse (50 μg/mL) (Sigma Aldrich, USA) treatment was given and finally stained with propidium iodide (10 μg/mL) (Sigma Aldrich, USA) and subjected to flow cytometry (Beckman Coulter, USA) using cell quest pro software, excitation at 488 nm laser and emission at 560/670 nm. DNA content of 10,000 cells was recorded per sample and histograms were analyzed by Flowing software (version 2.5) [19].

### Western blotting

Jurkat cells were seeded in 24 well plate at 0.5 x 10^5^ cells/ml of complete growth culture media, incubated and treated with test compound C02 for 12 h and 24 h. The cells were lysed in PBS containing 1% (v/v) NP40, 1 mM EDTA, 150 mM NaCl, protease and phosphatase inhibitors, and 10 mM Tris (pH 7.4) (Sigma Aldrich, USA). Samples were incubated on ice for 10 minutes, briefly sonicated, and centrifuged for 10 minutes at 15000g to remove insoluble cellular debris. Cell protein extract was then resolved by SDS polyacrylamide gel electrophoresis, transferred to a polyvinylidene difluoride (PVDF) membrane (GE Healthcare), blocked overnight in PBS containing 5% (w/v) skim milk powder, incubated for 2 h with primary antibody, and then incubated for 2 h with secondary antibody solutions. Mouse anti-phospho SR proteins mAb1H4 and rabbit anti-actin antibodies were used as primary antibodies. The secondary antibodies were anti-mouse peroxidase conjugated and anti-rabbit peroxidase-conjugated. Proteins bands were visualized using 3, 3’-Diaminobenzidine tetrahydrochloride As per the manufacturer’s protocol. ^19^Actin signals were used as a loading control.

### Quantitative gene expression analysis

Jurkat cells were treated with compound C02 for 12 h and 24 h and DMSO was used as vehicle control. After incubation, mRNA was extracted using RNeasy mini kit (Qiagen, Germany) and quantified by spectrophotometry and analyzed on 1% agarose gel electrophoresis. cDNA was prepared using cDNA synthesis kit (Qiagen, Germany) according to the manufacturer’s protocol. Further the qRT-PCR was performed using SYBR Green I dye. cDNAs was used as the template for amplifications following the manufacturer's protocols. All primers used in the assays are listed in Supplementary Table 3. The expressions of test genes were normalized by using Actin as an endogenous control.

### Statistical analysis of relative mRNA quantification and protein quantification

mRNA levels of target genes were determined relative to the endogenous control Actin, according to the formula 2 to the power of delta cycle threshold (2DCt), where DCt¼Ct, reference gene – Ct, test gene. Differences between experimental groups were tested for significance using nonparametric Mann–Whitney test (GraphPad Prism version 5, San Diego, CA), for both mRNA, protein expressions and other analysis. Levels of significance are indicated by p< 0.05.

## Supplementary Material

Supplementary Methods

Supplementary Figures

Supplementary Tables
