# Supplementary material for: Identification of a novel and potent small molecule inhibitor of SRPK1: mechanism of dual inhibition of SRPK1 for the inhibition of cancer progression
Source: Aging (Albany NY). 2020 Dec 3;13(1):163–80. doi: 10.18632/aging.202301 (PMC7835025; doi:10.18632/aging.202301)
Supplement: Supplementary Methods [file aging-13-202301-s001.pdf]

SUPPLEMENTARY METHODS

Ligand preparation

Ligprep of Schrodinger suite [1] was used for ligand preparation in which 2D SDF files were converted into 3-dimentional SDF format with correct chiralities. The tautomeric, ionization states were generated by Epik at 7.0 +2.0 pH units and valences were filled by hydrogen atoms to prevent covalent binding with the receptor. Geometry minimization was done using the OPLS 2005 force field and compounds were further filtered on the basis of geometrical parameters.

Protein preparation

The protein structure was prepared for docking studies using Protein Preparation Wizard in Maestro v9.2 [2]. The protein was preprocessed by removal of water molecules, addition of hydrogen atoms, assigning bond orders and termini capping. The heteroatom present in the co-crystal structure of SRPK1 complex with SERPIN340 (PDB ID: 4WUA) were deleted. The internal H-bond network was optimized using H-bond assignment tool. Further restrain of heavy atoms was performed by impref utility in the OPLS 2005 force field.

Grid generation

The grid enables to exclude the undesirable residue present near the receptor volume or docking site and which may interact with ligand. Receptor Grid Generation panel of Schrodinger suite was used and grid points were defined on the basis of specified residues at the binding pocket which were either involved in active site or interacting with ASF/SF2. The non covalent interactions like hydrogen bonds to aromatic hydrogens and halogens were considered and grid was generated in predefined settings under the OPLS 2005 force field.

Molecular docking

The Glide module [3] was used for molecular docking. 10,919 ligand structures entered the critical docking phase and at most 5 poses per ligand were considered, the generated grid helped is defining the receptor for screening of ligands. Monte Carlo-based algorithm was applied for the pose and score of each hit. The docking results were exported to maestro 9.5, the best pose and molecular descriptors of lead compounds, the interactions like H-Bond, π-π stacking, hydrophobic interactions, electrostatic interactions, and energy of the docking model were cumulatively considered for selection of the novel compounds.

Molecular dynamics simulations

For each system the topology of protein was generated using GROMOS 9653a6 force field, while ligands topology was generated using ProDRG server [4]. All the systems were solvated using Simple Point Charge (SPC) Model in a dodecahedron box. Moreover, amounts of Na^+ and Cl^- corresponding to a concentration of 0.1 M were added to the system to mimic the cellular environment using the genion tool. At the beginning stage, energy optimization was used to eliminate the system's atomic coordinates from overlapping by choosing the steepest descent method for maximum 5000 steps ($F_{max} < 100$ $kJ \cdot mol^{-1} \cdot nm^{-1}$). Longrange electrostatic forces were calculated by Particle Mesh Ewald method [5]. A 1.0 nm radius cut-off was considered for the computation of Lennard-Jones and Coulomb interactions. Then the position restraint simulation of 500ps was carried out under NVT and NPT conditions. Finally, all the systems were introduced to MDS. A 2fs interval was given for saving the coordinates. The root mean square deviation (RMSD), root mean square fluctuation (RMSF), radius of gyration (RG) and hydrogen bond (Hbond) were calculated using g_rms, g_rmsf, g_covar, g_hbond. The trajectories were analyzed by Visual Molecular Dynamics [6] and Chimera [7]. Origin [8] was employed for originating and visualizing the plots.

REFERENCES

1. Schrödinger, LLC, New York, N. LigPrep, version 2.5. Suite 2012 (2012).
2. L, S. Maestro, version 9.2. New York, NY. (2010).
3. Schrödinger Llc New York Ny. Glide, version 5.7. Glide Schrödinger LLC NY (2011).
4. Schüttelkopf AW, van Aalten DM. PRODRG: a tool for high-throughput crystallography of protein-ligand complexes. Acta Crystallogr D Biol Crystallogr. 2004; 60:1355–63. https://doi.org/10.1107/S0907444904011679 PMID:15272157
5. Essmann, U, Perera L, Berkowitz ML. A smooth particle mesh Ewald method. J Chem Phys. 1995; 103. https://doi.org/10.1063/1.470117
6. Humphrey W, Dalke A, Schulten K. VMD: Visual

molecular dynamics. J Mol Graph. 1996; 14:33–38. https://doi.org/10.1016/0263-7855(96)00018-5

7. Pettersen EF, Goddard TD, Huang CC, Couch GS, Greenblatt DM, Meng EC, Ferrin TE. UCSF Chimera–a visualization system for exploratory research and analysis. J Comput Chem. 2004; 25:1605–12. https://doi.org/10.1002/jcc.20084 PMID:15264254

8. Smith TJ, Stevenson KJ. Origin 7.5 OriginLab Corporation, One Roundhouse Plaza, Northampton, MA 01060. 1-800-969-7720. www.OriginLab.com. Suggested price $699.00 (retail, single user), $489.00 (educational, single user). Contact company for other pricing options. J. Am. Chem. Soc. (2004). https://doi.org/10.1021/ja040926x
