# Supplementary material for: Identification of a novel and potent small molecule inhibitor of SRPK1: mechanism of dual inhibition of SRPK1 for the inhibition of cancer progression
Source: Aging (Albany NY). 2020 Dec 3;13(1):163–80. doi: 10.18632/aging.202301 (PMC7835025; doi:10.18632/aging.202301)
Supplement: Supplementary Figures [file aging-13-202301-s002.pdf]

SUPPLEMENTARY FIGURES

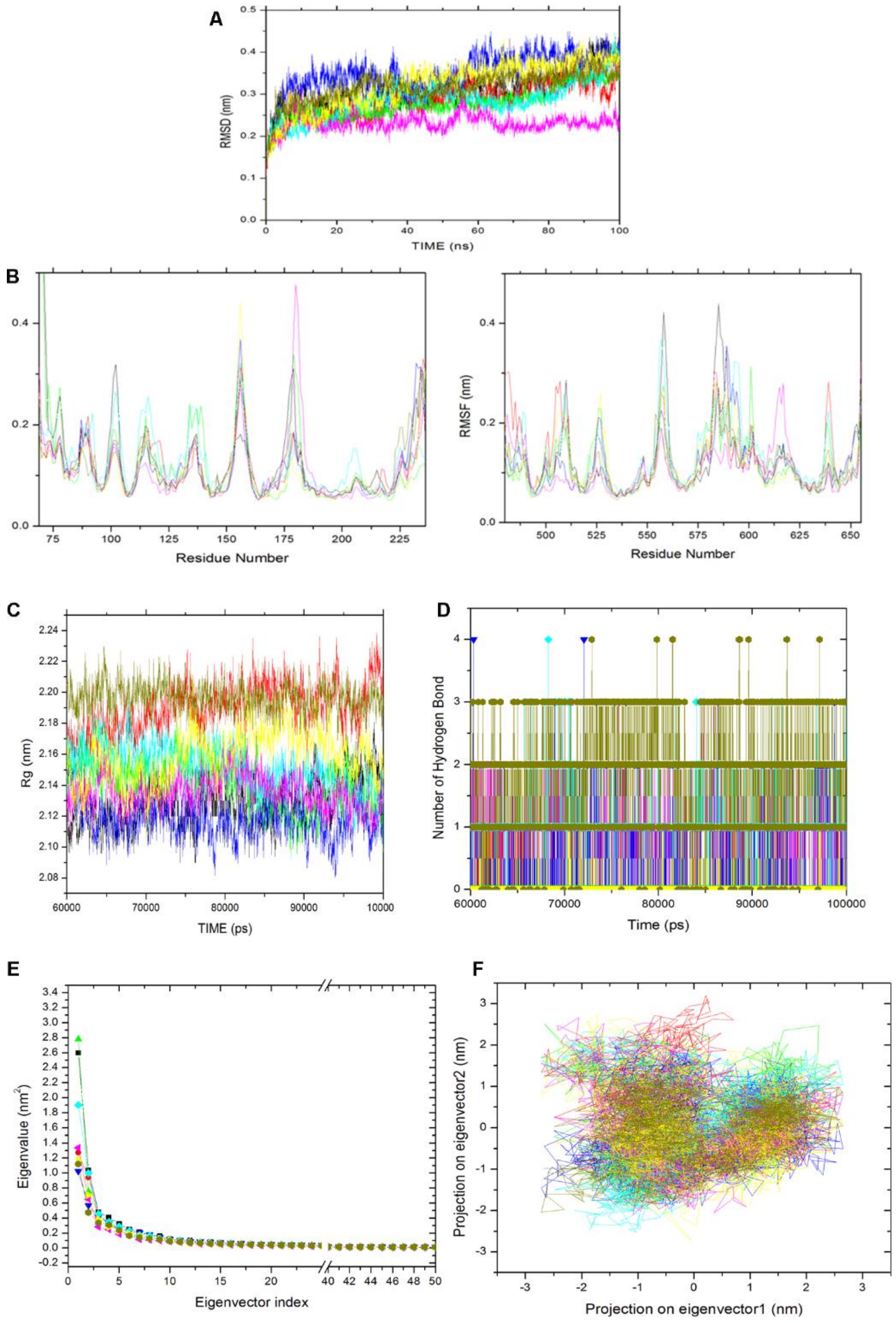

Supplementary Figure 1. Molecular dynamic simulations of apo SRPK1 and complexes. (**A**) RMSD of C$^{\alpha}$ backbone for SRPK1-Compound complexes as a function of time. (**B**) RMSF of residues during simulation. (**C**) Radius of gyration as a function of time (**D**) Number of hydrogen bonded interactions as a function of time. (**E**) Plot of eigen value vs. eigenvector index only first 50 out of 1000 are represented in the figure. (**F**) PCA scatter plot along first two principle components, PC1 and PC2 showing all atom fluctuations. Apo-SRPK1, SRPK1-SRPIN340, SRPK1-ZINC00518605 (C01), SRPK1-ZINC02154892 (C02), SRPK1-ZINC23127139 (C03), SRPK1-ZINC28182826 (C04), SRPK1-ZINC62001834 (C05), SRPK1-ZINC70666371 (C06) are shown in black, red, green, blue, cyan, pink, yellow, olive colors, respectively.

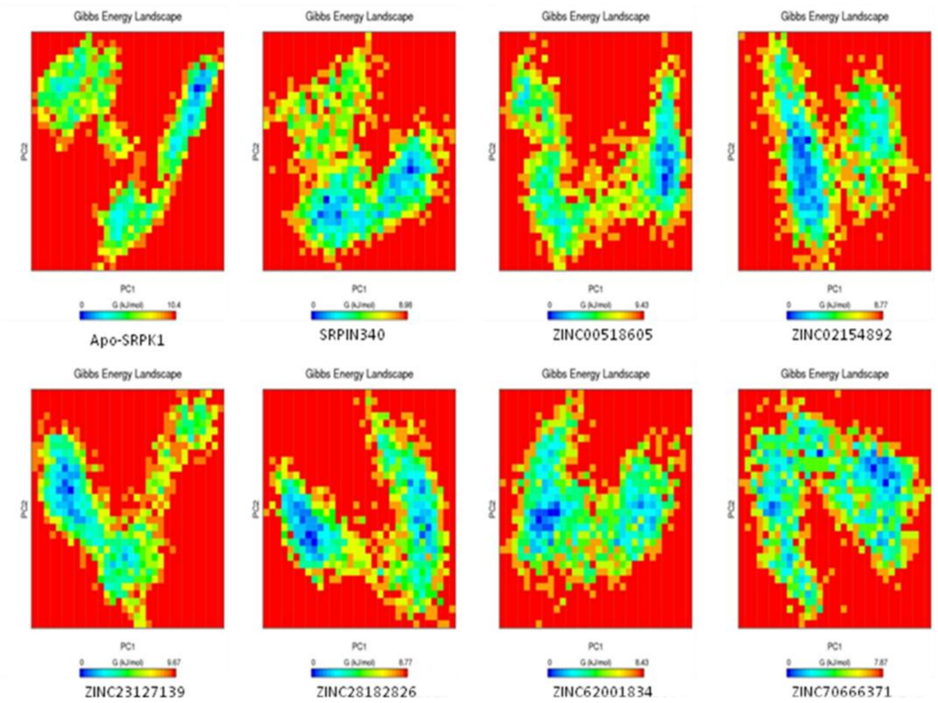

Supplementary Figure 2. The Gibbs energy landscape obtained during 100 ns MD simulations.
