# Supplementary material for: Identification of a novel and potent small molecule inhibitor of SRPK1: mechanism of dual inhibition of SRPK1 for the inhibition of cancer progression
Source: Aging (Albany NY). 2020 Dec 3;13(1):163–80. doi: 10.18632/aging.202301 (PMC7835025; doi:10.18632/aging.202301)
Supplement: Supplementary Tables [file aging-13-202301-s003.pdf]

SUPPLEMENTARY TABLES

Supplementary Table 1. QikProp analysis of physically remarkable descriptors and pharmaceutically admissible properties of screened compounds.

S.No	Compound Id	Compound name	mol MW	donorHB	accptHB	QPlogS	QPlogPo/w
1	ZINC00518605	C01	339.347	1	6.25	-4.683	3.237
2	ZINC02154892	C02	440.452	1.5	8.5	-4.069	1.819
3	ZINC23127139	C03	409.361	3	7.2	-2.66	2.297
4	ZINC28182826	C04	477.559	2	9.7	-3.102	2.629
5	ZINC62001834	C05	383.357	2	7.75	-4.789	1.817
6	ZINC70666371	C06	397.384	1	7.75	-5.22	2.609

Supplementary Table 2. Free binding energy calculations (kJ/mol^{-1}) of compounds using simulation trajectories.

Compound name	van der waal energy	Electrostatic energy	Polar solvation energy	SASA energy	Binding energy
C01	-218 ± 17	-19 ± 6	104 ± 13	-19 ± 1	-152 ± 5
C02	-168 ± 13	-34 ± 21	110 ± 27	-17 ± 1	-110 ± 14
C03	-228 ± 10	-16 ± 9	92 ± 13	-21 ± 1	-172 ± 14
C04	-259 ± 12	-16 ± 8	116 ± 18	-23 ± 1	-183± 18
C05	-212 ± 11	-17 ± 13	100 ± 16	-21 ± 1	-150 ± 14
C06	-245 ± 12	-41 ± 7	125 ± 10	-21 ± 1	-182 ± 12
SRPIN340	-174 ± 12	-5 ± 4	67 ± 10	-18 ± 1	-130 ± 13

Supplementary Table 3. List of primers used for qRT-PCR.

			Pro-apoptotic – apoptotic genes	
TNF-α	F	5′	AGGCGCTCCCCAAGAAGACA	3′
	R	5′	TCCTTGGCAAAACTGCACCT	3′
P53	F	5′	CACGAGCGCTGCTCAGATAGC	3′
	R	5′	ACAGGCACAAACACGCACAAA	3′
BAX	F	5′	TTCATCCAGGATCGAGCAGA	3′
	R	5′	GCAAAGTAGAAGGCAACG	3′
Cyt-C	F	5′	AGTGGCTAGAGTGGTCATTCATTTACA	3′
	R	5′	TCATGATCTGAATTCTGGTGTATGAGA	3′
APAF-1	F	5′	GATATGGAATGTCTCAGATGGCC	3′
	R	5′	GGTCTGTGAGGACTCCCCA	3′
Caspase 3	F	5′	GGTATTGAGACAGACAGTGG	3′
	R	5′	CATGGGATCTGTTTCTTTGC	3′
